# New Evidence on an old Question: a Meta-Analysis of Wallace versus Bricker Anastomoses

**DOI:** 10.1590/S1677-5538.IBJU.2025.0100

**Published:** 2025-06-20

**Authors:** Francisco Jazon de Araújo, Frank Robisom Costa de Sousa, Camille Rodrigues Aggensteiner, Gabriel Bruno Jácome de Melo, Pedro Aquiles Souza das Chagas, Thomas Silva de Queiroz, Rafael Paiva Arruda, Francisco Eugênio Vasconcelos, Paulo Silveira Campos Soares, Cristiano Araújo Costa, João Pompeu Frota Magalhães, Bárbara Vieira Lima Aguiar Melão

**Affiliations:** 1 Universidade Federal do Ceará Departamento de Nefrologia e Urologia Sobral Ceará Brasil Departamento de Nefrologia e Urologia, Universidade Federal do Ceará, Sobral, Ceará, Brasil; 2 Centro Universitário Inta Departamento de Urologia Sobral Ceará Brasil Departamento de Urologia, Centro Universitário Inta (Uninta), Sobral, Ceará, Brasil; 3 Santa Casa de Misericórdia de Sobral Departamento de Urologia Sobral Ceará Brasil Departamento de Urologia, Santa Casa de Misericórdia de Sobral, Sobral, Ceará, Brasil; 4 Hospital Adventista de Manaus Departamento de Oncologia Manaus Amazonas Brasil Departamento de Oncologia, Hospital Adventista de Manaus, Manaus, Amazonas, Brasil

**Keywords:** Urinary Bladder Neoplasms, Anastomosis, Surgical, Cystectomy

## Abstract

**Purpose:**

This meta-analysis compares the efficacy and safety of the Bricker and Wallace techniques, focusing on updating previously unassessed clinical outcomes to inform surgical decision-making.

**Material and Methods:**

A systematic review and meta-analysis followed PRISMA and Cochrane guidelines, with the protocol in PROSPERO (CRD42024621076). Searches in MEDLINE/PubMed, EMBASE, and Cochrane Library included Randomized Clinical Trials and cohort studies comparing both anastomosis techniques. Analyses used Odds Ratio (OR) and mean differences with a random-effects model.

**Results:**

Fourteen studies with 1,903 patients (980 Bricker; 923 Wallace) were included. No significant difference was found in overall stricture rates. However, the Bricker technique had more unilateral strictures (OR 0.47; 95% CI 0.30-0.75; p < 0.01), while the Wallace technique had lower stricture rates in patients who underwent ileal-conduit urinary diversion (OR 0.35; 95% CI 0.19-0.64; p < 0.001), and patients without prior radiotherapy (OR 0.29; 95% CI 0.14-0.61; p < 0.001). Wallace also presented reduced hydronephrosis (OR 0.37; 95% CI 0.17-0.79; p < 0.05). No significant differences were observed in patients undergoing neobladder diversion or those with bladder cancer.

**Conclusion:**

No difference in main analyses of stricture rates was found, supporting that technique choice should rely on surgeon preference and expertise. Therefore, beyond surgeon preference, the choice of technique should consider the patient's history of radiotherapy, and the type of urinary diversion planned, aiming to optimize postoperative outcomes and minimize the risk of specific complications.

## INTRODUCTION

Radical cystectomy (RC) is commonly performed on patients with muscle-invasive bladder cancer, non-urothelial malignancies, dysfunctional bladder, and chronic pelvic pain syndrome (1). Besides the emergence of innovative techniques and platforms in the management of these patients (2, 3), the type of surgical approach chosen, whether open, laparoscopic, or robot-assisted, may influence key operative outcomes (4). The life expectancy and subsequent morbidity and mortality rates of these patients depend, to a large extent, on the urinary diversion performed following the procedure (1). Over the years, several techniques for urinary diversion using intestinal segments (5) have been described in literature, each with its own advantages and disadvantages. (6) The most widely used approaches are the Bricker and Wallace methods (6).

The Bricker technique, described in the early 1950s, was one of the first methods for ureteroenteric anastomosis (UEA). In this approach, each ureter is individually connected to the intestinal segment in an end-to-side configuration using absorbable sutures (7). By contrast, the Wallace approach, which was delineated later, around the 1960s, involves an end-to-end anastomosis, where the two ureters are spatulated, joined side by side, and connected as a single unit to the end of the intestinal segment (8).

At the time, prominent surgeons who popularized the Wallace technique in the United Kingdom believed that this method offered several advantages over Bricker's approach. Specifically, the Wallace technique involves creating a single, large anastomosis, which was thought to reduce the risk of strictures and eliminate the need for separately closing the proximal end of the conduit (9). However, over time, no robust scientific evidence has demonstrated a clear superiority of one technique over the other. As a result, the choice of approach generally depends on the surgeon's preference and experience rather than on definitive differences in clinical outcomes (10).

A previously published meta-analysis (10) found no significant differences in the risk of stricture when comparing the two techniques; however, other potential outcomes were not evaluated. Since then, new studies have emerged, allowing for a reevaluation of the previous findings and the inclusion of additional outcomes in the analysis. Therefore, this meta-analysis aims to explore the main differences in the impact of outcomes between the two approaches, providing a more comprehensive understanding of their clinical implications.

## MATERIAL AND METHODS

This systematic review and meta-analysis were performed and reported following the Cochrane Collaboration Handbook of Systematic Review of Interventions and the Preferred Reporting Items for the Systematic Reviews and Meta-analysis (PRISMA) statement guidelines (11, 12). The protocol was preregistered in the International Prospective Register of Systematic Reviews (PROSPERO; CRD42024621076).

### Search Strategy

We systematically searched PubMed (MEDLINE), Embase, and Cochrane Central Register of Controlled Trials from inception to August 2024. The search strategy included the terms "Wallace", "Bricker", and "Urinary Diversion" in both "AND" and "OR" combinations to maximize the results obtained. The complete search strategy is detailed in Supplementary Methods 1. Two authors (F.R.C.S. and F.J.A-N.) independently screened titles and abstracts and fully evaluated the studies for eligibility. Duplicates identified through title, author, and journal comparison were removed prior to eligibility assessment. To identify additional literature, we conducted reference mining in the included studies. Discrepancies were settled in a discussion panel with a third author (C.R.A).

### Eligibility Criteria

We restricted the inclusion in this study to articles that met all the following eligibility criteria: (I) randomized clinical trials (RCTs) or cohort studies (prospective or retrospective); (II) studies that enrolled participants who underwent either the Bricker or Wallace anastomosis techniques; (III) studies reported any outcomes of interest. Our exclusion criteria were studies published solely as conference abstracts; reviews; studies that were not written in English; studies with mixed populations where the data for Bricker and Wallace techniques could not be clearly separated.

### Data extraction

Two authors (F.R.C.S and F.J.A-N.) conducted data extraction independently, following predefined search criteria and quality assessment. The data extracted included article characteristics (publication year, surgical indication, authors, country, follow-up), population characteristics (age, sex, BMI, intervention used, radiotherapy pelvic history), intervention characteristics (type of urinary diversion technique), and outcomes.

### Outcomes and definitions

The efficacy prespecified outcomes were ureteroenteric stricture rates; unilateral ureteroenteric stricture rates, considering strictures affecting only the left or right ureteral unit; and bilateral ureteroenteric stricture rates, considering strictures occurring simultaneously on both the left and right ureteral units; time to stricture (in months); length of stay (LOS); urinary leakage rates; mean operative time (in minutes). The safety outcomes analyzed included electrolyte disturbance rates; blood transfusion rates; ileus rates; hydronephrosis rates. The analyses were stratified by comparison between Bricker technique group and Wallace technique group.

## Statistical Analysis

All statistical analyses were performed using Review Manager 5.4.1. We employed Odds Ratio (OR) with 95% confidence interval (CI) as the measure of effect size to report binary outcomes. Mean Differences (MD) with 95% CI were used for continuous outcomes. Heterogeneity was assessed with the Cochran Q test and I² statistics. I^2^ ≥ 50% were considered significant for heterogeneity. We used the Restricted Maximum Likelihood random‐effects model (13). Sensitivity analyses, employing the leave-one-out approach, were conducted when significant heterogeneity was observed. We also performed a subgroup analysis based on patients without previous radiotherapy, patients undergoing ileal conduit urinary diversion, patients without previous radiotherapy who underwent ileal conduit urinary diversion, patients undergoing neobladder urinary diversion, and patients with bladder cancer.

### Quality Assessment

The risk of bias and quality assessment in non-randomized studies were evaluated with the Newcastle Ottawa Scale (NOS), which includes the following domains: selection, comparability, and exposure (14). Two independent authors (G.B.J.M and C.R.A) completed the risk of bias assessment. Disagreements were resolved through a consensus with a third author (F.J.A-N.). Studies scoring from 0 to 3, 4 to 6, and 7 to 9 were considered as low, moderate, and high quality, respectively.

## RESULTS

### Study Selection and Characteristics

**Figure 1:**
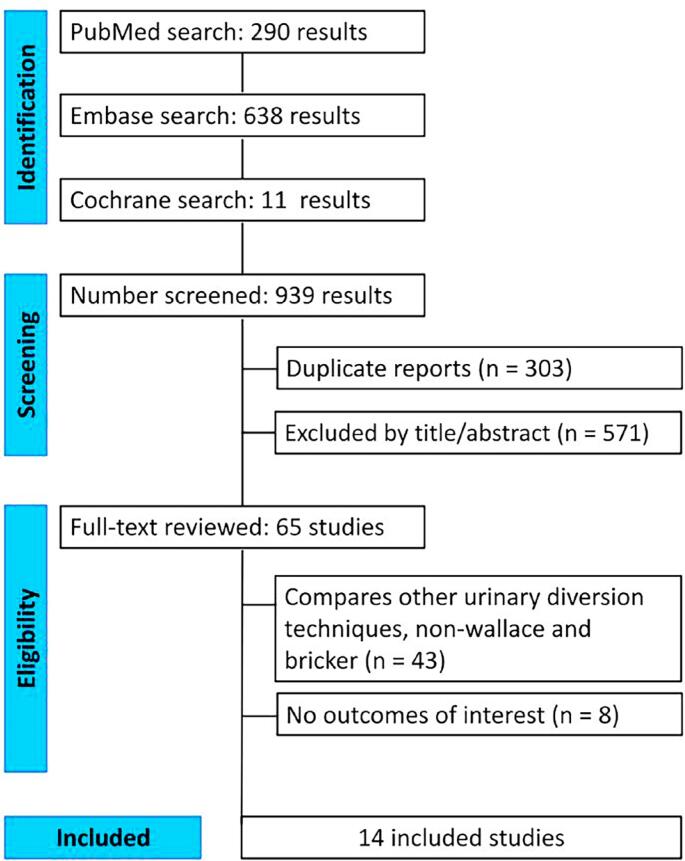
Diagram showing the study selection process according to PRISMA guidelines.

After a search of the literature, 939 studies were found. From these, 636 studies were eligible for title and abstract screening after duplicate removal. Out of these, 65 studies were eligible for full-text screening. Finally, 14 studies were included in this meta-analysis (Figure-1) (15–28).

The diagram illustrates the identification, screening, eligibility and inclusion of studies for the meta-analysis, resulting in 14 included studies.

A total of 1,903 patients underwent urinary diversion, with 980 (51.5%) in the Bricker technique group and 923 (48.5%) in the Wallace technique group. The mean age in the Bricker group was 66.13 years, compared to 61.89 years in the Wallace group. The most common surgical procedure preceding these anastomoses was RC performed for bladder cancer, with the ileal conduit being the predominant type of urinary diversion. Detailed baseline characteristics of the included studies are summarized in Table-1. More information is provided in Supplementary Methods 2.

**Table 1:** Baseline characteristics of included studies (Wallace / Bricker).

Study (Year)	Sample	Surgical indication	Male sex no. (%)	Mean / Median* age ± SD	Mean / Median* FU ± SD (months)	BMI ± SD	XRT pelvic history (%)
Adnan et al., 2022 (15)	43 / 73	RC for Bladder Cancer	33 (76.7) / 64 (87.7)	55 (± 11) / 63.66 (± 10.9)	48* (24-94)^R^	25.86 (± 4.89) / 26.31 (± 4.66)	8 (18.6) / 5 (6.8)
Al-Nader et al., 2021 (16)	209 / 209	RC for Bladder Cancer, cystectomy for others benign conditions or pelvic malignancies	172 (83.1) / 178 (86)	66.72 (± 9.3) / 66.9 (± 9)	25* (3-197)^R^ / 25* (3-85)^R^	26.2 (± 4.49) / 26.23 (± 4.62)	16 (7.7) / 13 (6.2)
Alhamdani et al., 2023 (28)	20 / 19	Radical cystectomy and radical cystoprostatectomy for bladder cancer	13 (65) / 18 (94.7)	67.5* (61.5–71)^IR^ / 67* (63–74)^IR^	12* (6.3–36)^IR^/ 19* (4–33.8)^IR^	NA	2 (10) / 0
Can et al., 2024 (17)	60 / 42	RC for Bladder Cancer	52 (86.7) / 37 (88.1)	66* (32–81)^R^ / 65* (40–75)^R^	20* (10–71)^R^ / 18* (10–31)^R^	27 / 26	Exclusion criteria
Christoph et al., 2019 (18)	65 / 75	RC for Bladder Cancer	46 (70.8) / 50 (66.7)	71* / 71*	17* / 36.5*	26.4 / 26.2	Exclusion criteria
Desai et al., 2014 (19)	86 / 46	RC for Bladder Cancer	114 (86.4)	60 (± 10)	25.1 (± 25.9)	26.8 (± 5.1)	NA
Djordjevic et al., 2021 (20)	30 / 30	RC with standard pelvic lymph node dissection	24 (80) / 22 (73.3)	68 (± 6.6) / 63 (± 7.2)	24	26.1 (± 3.2) / 27.2 (± 2.6)	Exclusion criteria
Evangelidis et al., 2006 (21)	112 / 86	Any patient undergoing Radical Cystectomy	78 (69.6) / 55 (64)	62 / 66	18.6 / 21.3	NA	14 (12.5) / 19 (22.1)
Kadoriku et al., 2024 (22)	32 / 23	Patients undergoing robotic-assisted intracorporeal ileal conduit urinary diversion	21 (65.6) / 18 (78.3)	73* (69–76)^IR^ / 77* (75–81)^IR^	12	22.8* (20.7–25.3)^IR^ / 24.2* (21.9–25.4)^IR^	0
Kouba et al., 2024 (23)	92 / 96	Cystectomy for bladder cancer	69 (75) / 75 (78.1)	66.7 (± 12.2) / 66.3 (± 11.9)	32.5 (± 21.4) / 34.3 (± 20.5)	25.9 (± 5.4) / 29.0 (± 6.3)	9 (10) / 15 (16)
Krafft et al., 2022 (24)	66 / 69	Cystectomy for any reason	48 (72.7) / 55 (69.7)	67.6 (± 9) / 66.6 (± 10.8)	16* (6–58)^R^ / 14* (6–39)^R^	27 (± 4.4) / 26.7 (± 5.4)	6 (9.1) / 3 (4.3)
Liu et al., 2014 (25)	46 / 53	Radical cystectomy for transitional cell carcinoma	38 (82.6) / 44 (83)	62.7 (± 8.6) / 61.9 (± 9.0)	26.3 (± 10) / 26.4 (± 10.2)	23.5 (± 1.3) / 23.3 (± 1.9)	6 (13) / 5 (9.4)
Alonso Mediavilla et al., 2022 (26)	47 / 108	Patients undergoing urinary diversion employing small bowel	NA	NA	NA	NA	NA
Wiederhorn; Roberts, 1974 (27)	15 / 51	Patients with malignant or benign disease undergoing urinary diversion	NA	NA	29.8 / 34.17	NA	19 (28.79)

NA = not available; RC = Radical Cystectomy; SD = Standard deviation; Xrt = Radiation therapy;

* = Median;

R Range;

IR = Interquartile Range; FU = follow-up period

### Stricture analyses

**Figure 2:**
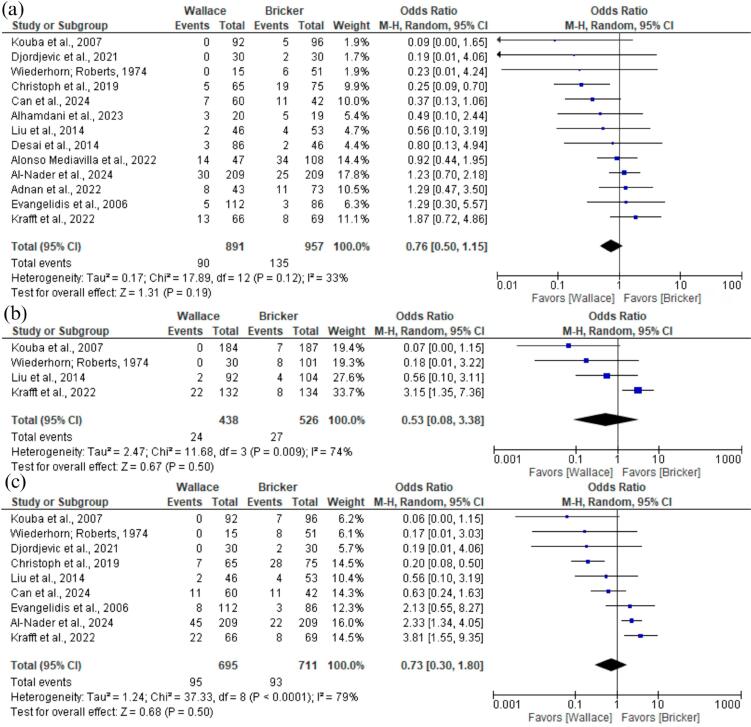
Forest plot of stricture rates.

The main analysis of stricture rates (patients with stricture relative total patients) was not significantly different between Bricker and Wallace techniques (OR 0.76; 95% CI 0.50 to 1.15; p = 0,19; I² =33%; Figure-2a). The analysis also indicated no statistical difference between groups when compared the occurrence of ureteral strictures relative to the total number of ureteral units (OR 0.53; 95% CI 0.08 to 3.38; p = 0,50; I² =74%; Figure-2b) and the occurrence of ureteral strictures relative to the total patient count (OR 0.73; 95% CI 0.30 to 1.80; p = 0,50; I² =79%; Figure-2c).

Forest plot for stricture rates in (a) patients with stricture per total patients, (b) ureteral stricture per total ureter units and (c) ureteral stricture per total patients.

**Figure 3:**
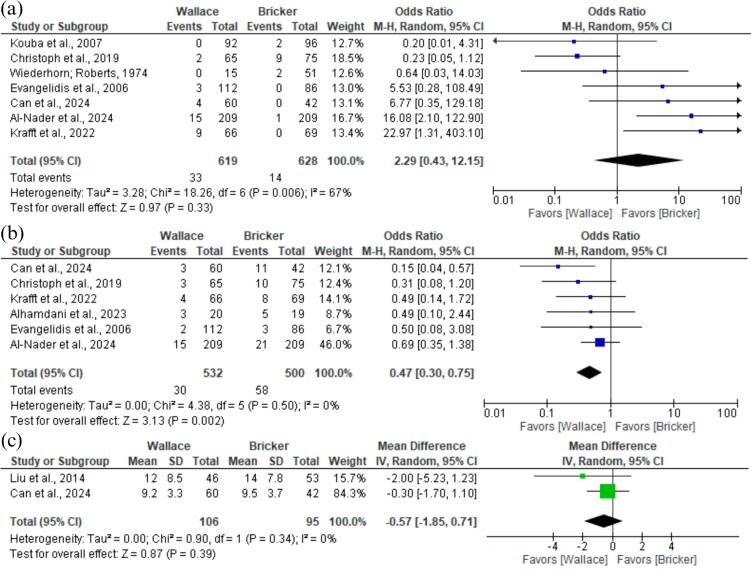
Forest plot of bilateral stricture, unilateral stricture and time to stricture (in months).

The analysis indicated no significant difference on bilateral stricture between Wallace group and Bricker group (OR 2.29; 95% CI 0.43 to 12.15; p = 0,33; I² =67%; Figure-3a), while in the Bricker technique there was a significant increase of unilateral stricture (OR 0.47; 95% CI 0.30 to 0.75; p<0,01; I² =0%; Figure-3b). The analysis of the median time to stricture revealed no significant difference across these groups (MD −0.57; 95% CI −1.85 to 0.71; p = 0,39; I² =0%; Figure-3c).

Forest plot for (a) patients with bilateral stricture per total patients, (b) patients with unilateral strictrure per total patients and (c) median time to stricture, in months.

### Other Efficacy Outcomes

**Figure 4:**
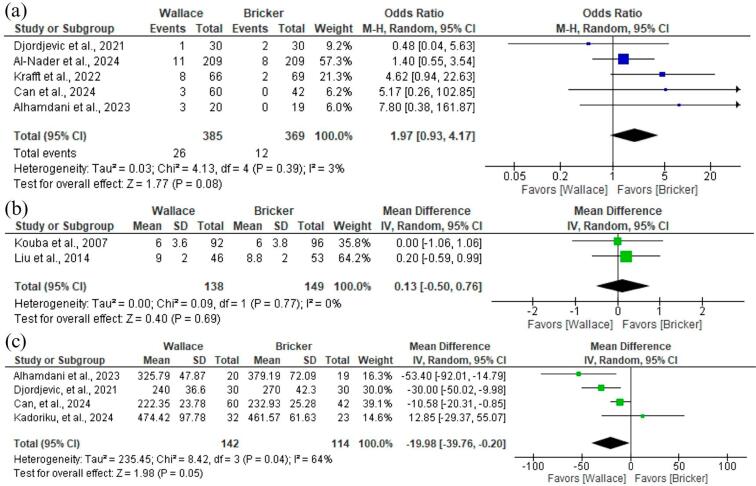
Forest Plot of Urinary Leak, LOS and Mean Operative Time.

Our findings indicated that there was no significant difference in urinary leakage between the Bricker group and the Wallace group (OR 1.97; 95% CI 0.93 to 4.17; p = 0,08; I²=3%; Figure-4a). Likewise, LOS presented no significant difference between the techniques (MD 0.13; 95% CI −0.50 to 0.76; p = 0,69; I² =0%; Figure-4b). A pooled analysis of four studies, including 256 patients, showed that the Wallace group had a reduced operative time than the Bricker group (MD −19.98; 95% CI −39.76 to −0.20; p = 0,05; I² =64%%; Figure-4c).

Forest plot for (a) urinary leakage occurrence, (b) LOS and (c) mean operative time, in minutes.

### Safety outcomes

**Figure 5:**
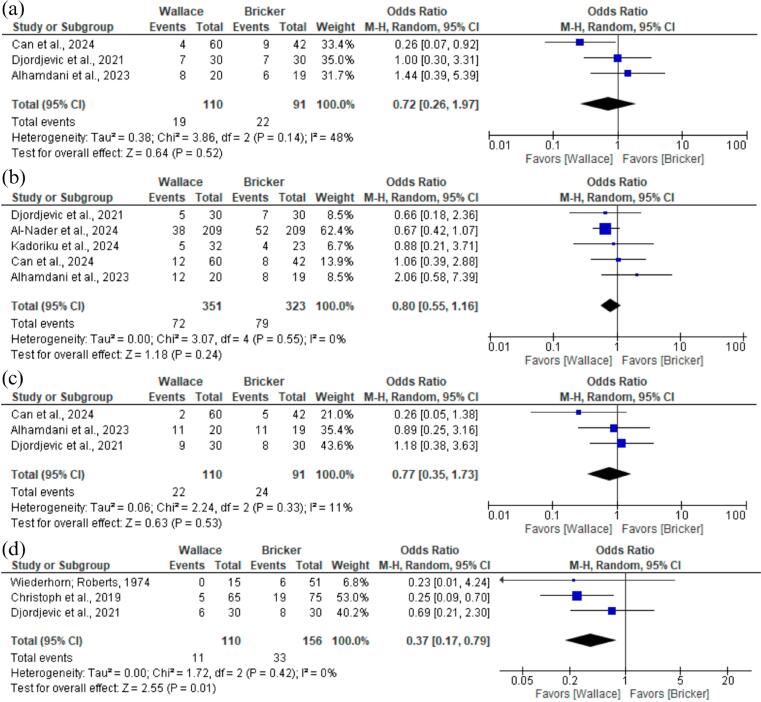
Forest plot of safety outcomes.

We also examined safety outcomes related to surgery and postoperative complications such as electrolyte disturbance (OR 0.72; 95% CI 0.26 to 1.97; p = 0,52; I² =48%; Figure-5a), blood transfusion (OR 0.80; 95% CI 0.55 to 1.16; p = 0,24; I² =0%; Figure-5b), ileus (OR 0.77; 95% CI 0.35 to 1.73; p = 0,53; I² =11%; Figure-5c). All of these analyses showed no significant difference between Wallace or Bricker groups. However, the meta-analysis of three studies regarding hydronephrosis showed better results for the Wallace group (OR 0.37; 95% CI 0.17 to 0.79; p<0,05; I² =0%; Figure-5d).

Forest plot for (a) electrolyte disturbance rates, (b) blood transfusion rates, (c) ileus rates and (d) hydronephrosis rates.

### Subgroup analyses of Stricture rates

**Figure 6:**
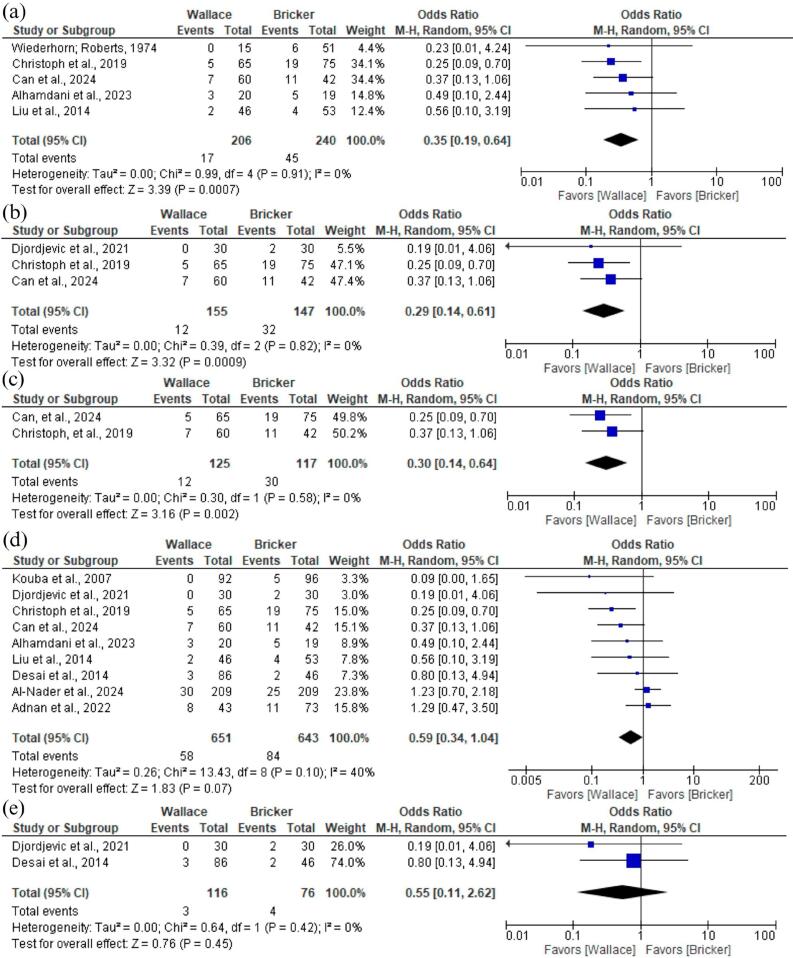
Forest plot of subgroup analysis.

We conducted a subgroup analysis comparing stricture on different populations. Furthermore, the Wallace technique revealed significantly lower stricture rates compared to Bricker on patients who underwent ileal-conduit urinary diversion (OR 0.35; 95% CI 0.19 to 0.64; p < 0,001; I² =0%; Figure 6a), in those not previously subjected to radiotherapy (OR 0.29; 95% CI 0.14 to 0.61; p < 0,001; I² =0%; Figure-6b), and a combined analysis of both these subgroups (OR 0.30; 95% CI 0.14 to 0.64; p < 0,01; I² =0%; Figure-6c). There was no significant difference between these techniques in the patients diagnosed with bladder cancer (OR 0.59; 95% CI 0.34 to 1.04; p = 0,07; I² =40%; Figure-6d), and in patients who underwent neobladder urinary diversion (OR 0.55; 95% CI 0.11 to 2.62; p = 0,45; I² =0%; Figure 6e). The summary of findings in this meta-analysis are shown in Table-2.

Forest plots for patients with stricture per total patients in (a) patients who underwent ileal-conduit urinary diversion, (b) those not previously subjected to radiotherapy, (c) patients without previous radiotherapy who underwent ileal conduit urinary diversion, (d) patients diagnosed with bladder cancer and (e) patients who underwent neobladder urinary diversion.

**Table 2:** Pooled outcomes of Wallace urinary diversion compared to Bricker urinary diversion.

Outcomes and subgroup analysis	Effect Size (95% CI)	P value	I²	No. of Studies	No. of Patients (Wallace vs Bricker)
Stricture rates (patients with stricture relative total patients)	OR 0.76 (0.50-1.15)	0,19	33%	13	891 vs 957
Stricture rates (ureteral stricture relative total ureter units)	OR 0.53 (0.08-3.38)	0,5	74%	4	438 vs 526
Stricture rates (ureteral stricture relative total patients)	OR 0.73 (0.30-1.80)	0,5	79%	9	695 vs 711
Bilateral stricture	OR 2.29 (0.43-12.15)	0,33	67%	7	619 vs 628
Unilateral strictures	OR 0.47 (0.30-0.75)	<0,01	0%	6	532 vs 500
Median time to stricture (in months)	MD −0.57 (-1.85-0.71)	0,39	0%	2	106 vs 95
Urinary leakage occurrence	OR 1.97 (0.93-4.17)	0,08	3%	5	385 vs 369
Length of Stay	MD 0.13 (-0.50-0.76)	0,69	0%	2	138 vs 149
Mean operative time (in minutes)	MD −19.98 (-39.76--0.20)	0,05	64%	4	142 vs 114
Electrolyte disturbance rates	OR 0.72 (0.26-1.97)	0,52	48%	3	110 vs 91
Blood transfusion rates	OR 0.80 (0.55-1.16)	0,24	0%	5	351 vs 323
Ileus rates	OR 0.77 (0.35-1.73)	0,53	11%	3	110 vs 91
Hydronephrosis rates	OR 0.37 (0.17-0.79)	<0,05	0%	3	110 vs 156
Stricture rates in patients who underwent ileal-conduit urinary diversion	OR 0.35 (0.19-0.64)	<0,001	0%	5	206 vs 240
Stricture rates in patients not previously subjected to radiotherapy	OR 0.29 (0.14-0.61)	<0,001	0%	3	155 vs 147
Stricture rates in patients without previous radiotherapy who underwent ileal conduit urinary diversion	OR 0.30 (0.14-0.64)	<0,01	0%	2	125 vs 117
Stricture rates in patients diagnosed with bladder cancer	OR 0.59 (0.34-1.04)	0,07	40%	9	651 vs 643
Stricture rates in patients who underwent neobladder urinary diversion	OR 0.55 (0.11-2.62)	0,45	0%	2	116 vs 76

OR = odds ratio; MD = Mean difference; No. = Number.

### Sensitivity analyses

The occurrence of ureteral strictures relative to the total number of ureteral units presented a high heterogeneity. The heterogeneity was resolved after excluding Krafft et al. (I^2^ = 0 %), with a significant result favoring the Wallace group (Supplementary Methods 4). Even after the leave-one-out approach, heterogeneity remained high when considering the occurrence of ureteral strictures relative to the total patient count (I² = 65%) (Supplementary Methods 5). For the bilateral stricture rates, the omission of the study Christoph et al. reduced the heterogeneity present to 40% (Supplementary Methods 6), yet the outcome still showed no difference between Wallace and Bricker groups. Finally, after performing a leave-one-out sensitivity analysis, the Wallace technique no longer maintained its superiority over Bricker, while heterogeneity remained considerable (I² = 55%) (Supplementary Methods 7).

### Quality assessment

A summary of the risk of bias assessment is provided in Supplementary Methods 3. Of the fourteen included studies, seven had a low risk of bias, six were evaluated as moderate, and one had a high risk of bias. The outcome domain showed strong performance, with most studies employing robust methods to assess outcomes and ensuring sufficient follow-up time. Overall, the studies exhibited high or moderate scores across all domains.

## DISCUSSION

In this systematic review and meta-analysis of 14 studies and 1,903 patients, we evaluated the comparative efficacy and safety between patients who underwent urinary diversion using the Wallace or Bricker technique. One of the key findings was that: (I) both techniques demonstrated similar results regarding main analyses of stricture rates (patients with stricture relative total patients; ureteral stricture relative total ureter units; and ureteral stricture relative total patients). Additionally, to our knowledge, this is the first meta-analysis to compare efficacy and safety outcomes, excluding stricture rates, between the Bricker and Wallace techniques. The findings listed below represent novel contributions to the field: (I) there were fewer occurrences of unilateral strictures, and the operative time was shorter in the Wallace group; (II) we showed no significant differences between the techniques in terms of bilateral stricture, urinary leakage, median time to stricture, LOS, ileus, hydronephrosis, electrolyte disturbance, blood transfusion; (III) the subgroup analysis showed that the Wallace technique was favored in patients who underwent ileal-conduit urinary diversion and patients not previously subjected to radiotherapy, and patients with both conditions simultaneously; (IV) no significant differences were observed between the techniques in patients diagnosed with bladder cancer or patients who underwent neobladder urinary diversion.

Bladder replacement with an ileal conduit is a common procedure following cystectomy for bladder cancer (29). Bricker (7) standardized this technique, while Wallace (8) later proposed a modification that involves joining the ureters into a single confluence before attaching them to the conduit. Although Wallace's approach is considered more time-efficient (16), the Bricker technique remains the preferred choice for many surgeons, largely due to familiarity and personal preference (6). A previous meta-analysis, however, has not demonstrated a clear advantage of one technique over the other (10). One of the first comparative studies, conducted in 1974, suggested a lower risk of postoperative complications with Wallace's technique, though potential bias exists due to imbalanced sample sizes (27). However, to this day, the lack of randomized studies on the subject remains a significant limitation, hindering a reliable comparison between these techniques, as retrospective studies are inherently more prone to bias (11).

Although the pathophysiology of strictures remains unclear, it is a critical factor in determining postoperative outcomes, as it can significantly impact patient quality of life (30). The etiology of strictures varies, with technical errors and tissue edema being common causes (31). In addition, a previous meta-analysis (10), suggested that the choice of technique may not significantly influence its specific outcome. Similarly, our analyses revealed no statistically significant differences in the overall stricture rates between the Bricker and Wallace techniques, consistent across various metrics. First, the proportion of patients with strictures offers a clear and straightforward interpretation of the frequency of complications. Second, the incidence of ureteral strictures relative total ureter units allows for a more detailed evaluation by considering the number of ureteral units at risk. Lastly, the occurrence of ureteral strictures relative to total patients offers a global perspective of the techniques impact of the entire patient population. The use of three distinct metrics strengthens the evidence by offering a more comprehensive analysis.

Nevertheless, the equivalence in overall stricture rates reinforces the notion that the choice of technique should be primarily guided by the surgeon's preference and expertise, as well as tailored to the clinical features of each individual patient (25). However, despite the persistence of high heterogeneity in the sensitivity analysis of ureteral strictures relative to total patients, the analysis based on total ureter units showed significantly lower stricture rates in the Wallace group. While this finding suggests a potential advantage of the Wallace technique, the observational design of the included studies limits the ability to draw firm conclusions regarding causality.

The analysis of the laterality of stenosis highlights the importance of understanding the fundamental principles of each technique. While earlier concerns regarding bilateral strictures in the Wallace technique (24) or higher unilateral stricture rates in the Bricker technique have been noted in smaller studies (23), our results showed the slightly higher incidence of unilateral strictures observed in Bricker compared to Wallace. These findings align with the results of previous retrospective studies, as all studies included in this analysis reported a higher number of patients with unilateral strictures in the Bricker group than Wallace group (17, 18, 21, 24, 28). In the analysis of bilateral strictures, some studies suggest that the Wallace technique might result in a higher incidence of bilateral strictures compared to the Bricker technique (16, 22-25). However, we found no significant difference in the incidence of bilateral strictures between the two techniques, including in the sensitivity analysis. Furthermore, our study demonstrated no significant difference in the median time to stricture formation between the groups, consistent with findings reported in the literature (17, 32).

Urinary leakage is recognized as a risk factor for the development of ureteroenteric strictures (UES) and is one of the primary postoperative complications associated with urinary diversion (33). In our study, no significant difference in urinary leakage rates was observed between the techniques. Electrolyte disturbances and urine stasis may be associated with modified Bricker techniques or other approaches that involve a larger intestinal segment in an attempt to reduce UES rates (17). In addition, patients undergoing urinary diversion, and with advanced pelvic malignancies, appear to experience greater surgical complexity, a higher incidence of hydronephrosis, and an increased risk for UES development (16). Likely, in our analysis, the Wallace technique demonstrated significantly greater safety for hydronephrosis, aligning with findings from a study conducted in 1974 (27).

Regarding the complexity of selecting the UEA type, the studies included in this analysis consistently showed that the choice of technique was strongly influenced by the surgeon's personal preference and expertise, a pattern also supported by previous meta-analysis (10). Additionally, when performing cystectomies and urinary diversions, institutional factors specific to each center may impact operative outcomes (34, 35). In this context, one of the outcomes known to be influenced by institutional volume and experience is operative time (34), which, in relation to the choice of UEA technique, was shorter in the Wallace group according to our analysis (17, 20, 22, 28). Furthermore, the sensitivity analysis of this outcome revealed no significant difference between the techniques (17, 20, 22), with consistently high heterogeneity remaining unresolved. On the other hand, in the current study, the LOS was comparable, with no significant differences between the two techniques across all included studies (17, 23, 25, 28). Similarly, other outcomes, such as blood transfusion rates (16, 17, 20, 22, 28) and ileus incidence (17, 20, 28), showed no significant differences.

Additionally, our subgroup analyses provide a more in-depth examination of patient-specific factors, such as the influence of prior radiotherapy history, previous diagnoses, and the type of urinary diversion, which were not explored in previous meta-analysis (10). Despite the absence of clear recommendations regarding the ideal type of urinary diversion following cystectomy for bladder cancer (17) and the two established UEA techniques (10, 17) discussed in this study, our findings suggest no advantage of one technique over the other in these patients. Regarding urinary diversion types, the ileal conduit has been recognized for decades as one of the most widely adopted gold-standard techniques worldwide (22, 36). When analyzing this subgroup in the present study, our results found a lower likelihood of UES development when the Wallace technique was used in association with the ileal conduit. This finding holds critical importance, as the ileal conduit remains one of the most performed urinary diversions in clinical practice and is closely associated with patient outcomes (37). Conversely, in patients undergoing neobladder urinary diversion, a technique known for providing superior functional outcomes and quality of life (1), despite its more complex surgical execution and higher complication rates (19); in our analysis no significant differences were observed between the Bricker and Wallace techniques.

Moreover, a prior meta-analysis reported no difference in UES rates among patients with or without history of radiation therapy (10). Our results found that studies excluding patients with a history of radiotherapy demonstrated significantly lower UES rates when using the Wallace technique for UEA, with more stricture rates in the Bricker group. Additionally, supporting this observation, the subgroup analysis restricted patients without previous radiotherapy who underwent ileal conduit urinary diversion also favored the Wallace group. These findings underscore the importance of tailoring surgical techniques to a patient's specific needs and clinical context; more precisely, the selection of the method should consider the planned urinary diversion and the patient's prior radiotherapy, rather than applying a one-size-fits-all approach.

This systematic review and meta-analysis have limitations that should be acknowledged: (I) the absence of RCTs among the included studies represents a significant limitation, as observational and retrospective cohort designs are more susceptible to bias; (II) among the included studies, we identified differences in patient populations, including variations in diagnoses, disease severity and preoperative conditions, which may have influenced the reported outcomes; (III) small sample size in specific subgroups, such as patients undergoing neobladder urinary diversion, reduced the robustness of subgroup analyses; (IV) differences in surgical outcomes may reflect variability in surgeon expertise or institutional protocols, factors that were not consistently reported or controlled; (V) the criteria for allocating participants to the Wallace and Bricker groups varied among the included studies, potentially influencing reported outcomes; and (VI) late complications might have been underrepresented, given the relatively short follow-up of many included studies.

## CONCLUSIONS

Our findings indicate no significant differences in main analyses of stricture rates, median time to stricture, LOS, urinary leakage, bilateral stricture, electrolyte disturbances, blood transfusion, and ileus between the techniques examined, suggesting that the choice of approach should primarily be guided by the surgeon's judgment, experience, and the patient's unique clinical profile. However, in the laterality analysis, unilateral strictures appear to be more commonly associated with the Bricker technique, since the Wallace technique exhibited lower rates of UES. The Wallace technique also demonstrated advantages, including lower rates of UES in patients who underwent ileal conduit urinary diversion, without a history of radiotherapy and reduced rates of hydronephrosis.

For clinical practice, our subgroup results provide valuable information for individualized surgical selection. Therefore, beyond surgeon preference, the choice of technique should consider the patient's history of radiotherapy, and the type of urinary diversion planned, aiming to optimize postoperative outcomes and minimize the risk of specific complications. Recognizing the limitations of this study, larger and higher-quality randomized trials are necessary to more comprehensively evaluate the efficacy and safety of the Bricker and Wallace techniques.

## Figures and Tables

**Supplementary Methods 4:**
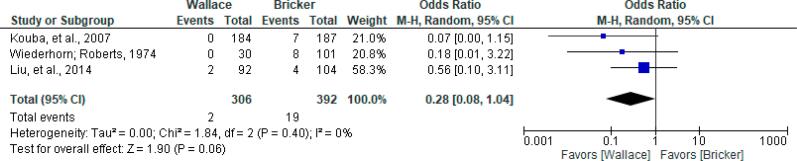
Sensitivity analysis of Ureteral strictures relative to the total number of ureteral units.

**Supplementary Methods 5:**
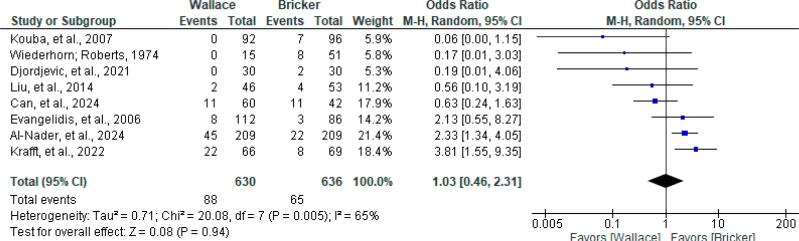
Sensitivity analysis of Ureteral strictures relative to the total patient count.

**Supplementary Methods 6:**
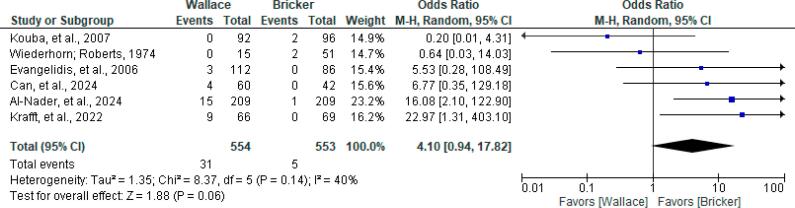
Sensitivity analysis of Bilateral stricture.

**Supplementary Methods 7:**
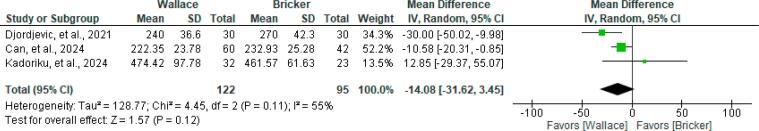
Sensitivity analysis of Mean operative time.

## ABBREVIATION LIST

BMI: = Body Mass Index
CI: = Confidence Interval
LOS: = Length of Stay
MD: = Mean Differences
OR: = Odds Ratio
PRISMA: = Preferred Reporting Items for Systematic Reviews and Meta-Analysis
PROSPERO: = International prospective register of systematic reviews
RC: = Radical Cystectomy
RCT: = Randomized Clinical Trials
SD: = Standard deviation
UEA: = Ureteroenteric Anastomosis
UES: = Ureteroenteric Stricture

## APPENDIX

**SUMMARY OF CONTENTS**
Supplementary Methods 1. Details of the search strategy according to the database 19
Supplementary Methods 2. Baseline characteristics of included studies (Wallace / Bricker) 20
Supplementary Methods 3. Newcastle-Ottawa Scale (NOS) assessment of non-randomized trials 21
Supplementary Methods 4. Sensitivity analysis of Ureteral strictures relative to the total number of ureteral units 22
Supplementary Methods 5. Sensitivity analysis of Ureteral strictures relative to the total patient count 22
Supplementary Methods 6. Sensitivity analysis of Bilateral stricture 22
Supplementary Methods 7. Sensitivity analysis of Mean operative time 23

**Supplementary Methods 1:** Details of the search strategy according to the database.

Database	Search strategy
PubMed/ MEDLINE	("Wallace" AND "Urinary Diversion"[mh]) OR ("Bricker" AND "Urinary Diversion"[mh]) OR ("Wallace" AND "Bricker")
Embase	(‘wallace’/exp OR ‘wallace’) AND (‘urinary diversion’/exp OR ‘urinary diversion’) OR (‘bricker’ AND (‘urinary diversion’/exp OR ‘urinary diversion’)) OR ((‘wallace’/exp OR ‘wallace’) AND ‘bricker’)
Cochrane	("Wallace" AND "Urinary Diversion") OR ("Bricker" AND "Urinary Diversion") OR ("Wallace" AND "Bricker")

**Supplementary Methods 2:** Baseline characteristics of included studies (Wallace / Bricker).

Study (Year)	Country	Sample	Surgical indication	Types of urinary diversion	Male sex no. (%)	Mean / Median* age ± SD	Mean / Median* FU ± SD (months)	BMI ± SD	XRT pelvic history (%)
Adnan et al., 2022 (15)	Pakistan	43 / 73	RC for Bladder Cancer	Ileal conduit and Orhotopic neobladder	33 (76.7) / 64 (87.7)	55 (± 11) / 63.66 (± 10.9)	48* (24-94)^R^	25.86 (± 4.89) / 26.31 (± 4.66)	8 (18.6) / 5 (6.8)
Al-Nader et al., 2021 (16)	Germany	209 / 209	RC for Bladder Cancer, cystectomy for others benign conditions or pelvic malignancies	Ileal conduit, Orthotopic bladder substitution or Continent cutaneous diversion	172 (83.1) / 178 (86)	66.72 (± 9.3) / 66.9 (± 9)	25* (3-197)^R^ / 25* (3-85)^R^	26.2 (± 4.49) / 26.23 (± 4.62)	16 (7.7) / 13 (6.2)
Alhamdani et al., 2023 (28)	Australia	20 / 19	Radical cystectomy and radical cystoprostatectomy for bladder cancer	Ileal conduit	13 (65) / 18 (94.7)	67.5* (61.5–71)^IR^ / 67* (63–74)^IR^	12* (6.3–36)^IR^ / 19* (4–33.8)^IR^	NA	2 (10) / 0
Can et al., 2024 (17)	Turkey	60 / 42	RC for Bladder Cancer	Ileal conduit	52 (86.7) / 37 (88.1)	66* (32–81)^R^ / 65* (40–75)^R^	20* (10–71)^R^ / 18* (10–31)^R^	27 / 26	Exclusion criteria
Christoph et al., 2019 (18)	Germany	65 / 75	RC for Bladder Cancer	Ileal conduit	46 (70.8) / 50 (66.7)	71* / 71*	17* / 36.5*	26.4 / 26.2	Exclusion criteria
Desai et al., 2014 (19)	Sweden/ USA	86 / 46	RC for Bladder Cancer	Robotic intracorporeal ileal neobladder	114 (86.4)	60 (± 10)	25.1 (± 25.9)	26.8 (± 5.1)	NA
Djordjevic et al., 2021 (20)	Serbia	30 / 30	RC with standard pelvic lymph node dissection	Hautmann neobladder with single chimney and Bricker / Hautmann neobladder with chimney modification and Wallace	24 (80) / 22 (73.3)	68 (± 6.6) / 63 (± 7.2)	24	26.1 (± 3.2) / 27.2 (± 2.6)	Exclusion criteria
Evangelidis et al., 2006 (21)	USA	112 / 86	Any patient undergoing Radical Cystectomy	Ileal conduit, Continent urinary reservoir or Neobladder	78 (69.6) / 55 (64)	62 / 66	18.6 / 21.3	NA	14 (12.5) / 19 (22.1)
Kadoriku et al., 2024 (22)	Japan	32 / 23	Patients undergoing robotic-assisted intracorporeal ileal conduit urinary diversion	robot-assisted intracorporeal ileal conduit urinary diversion	21 (65.6) / 18 (78.3)	73* (69–76)^IR^ / 77* (75–81)^IR^	12	22.8* (20.7–25.3)^IR^ / 24.2* (21.9–25.4)^IR^	0
Kouba et al., 2024 (23)	USA	92 / 96	Cystectomy for bladder cancer	Ileal conduit or Ileal neobladder	69 (75) / 75 (78.1)	66.7 (± 12.2) / 66.3 (± 11.9)	32.5 (± 21.4) / 34.3 (± 20.5)	25.9 (± 5.4) / 29.0 (± 6.3)	9 (10) / 15 (16)
Krafft et al., 2022 (24)	Germany	66 / 69	Cystectomy for any reason	Ileal conduit, Orthotopic bladder substitute or Continent cutaneous diversion	48 (72.7) / 55 (69.7)	67.6 (± 9) / 66.6 (± 10.8)	16* (6–58)^R^ / 14* (6–39)^R^	27 (± 4.4) / 26.7 (± 5.4)	6 (9.1) / 3 (4.3)
Liu et al., 2014 (25)	China	46 / 53	Radical cystectomy for transitional cell carcinoma	Ileal conduit	38 (82.6) / 44 (83)	62.7 (± 8.6) / 61.9 (± 9.0)	26.3 (± 10) / 26.4 (± 10.2)	23.5 (± 1.3) / 23.3 (± 1.9)	6 (13) / 5 (9.4)
Alonso Mediavilla et al., 2022 (26)	Spain	47 / 108	Patients undergoing urinary diversion employing small bowel	Ileal conduit or Neobladder	NA	NA	NA	NA	NA
Wiederhorn; Roberts, 1974 (27)	USA	15 / 51	Patients with malignant or benign disease undergoing urinary diversion	Ileal conduit	NA	NA	29.8 / 34.17	NA	19 (28.79)

NA = not available; RC = Radical Cystectomy; SD = Standard deviation; Xrt = Radiation therapy;

* = Median;

R = Range;

IR = Interquartile Range; FU = Follow-up period.

**Supplementary Methods 3:** Newcastle-Ottawa Scale (NOS) assessment of non-randomized trials.

Study	Selection				Comparability		Outcome			Total (9/9)
	Representative of the exposed cohort	Selection of external control	Ascertain ment of exposure	Outcome of interest not present at the start of the study	Main factor	Additional factor	Assessment of outcomes	Sufficient follow-up time	Adequacy of follow-up	
Adnan et al., 2022 (15)		^*^	^*^	^*^			^*^	^*^		5/9
Al-Nader et al., 2021 (16)		^*^	^*^	^*^			^*^	^*^		5/9
Alhamdani et al., 2023 (28)		^*^	^*^	^*^	^*^		^*^	^*^		6/9
Can et al., 2024 (17)		^*^	^*^	^*^	^*^	^*^	^*^	^*^		7/9
Christoph et al., 2019 (18)		^*^	^*^	^*^	^*^		^*^	^*^		6/9
Desai et al., 2014 (19)		^*^	^*^	^*^	^*^		^*^	^*^		6/9
Djordjevic et al., 2021 (20)		^*^	^*^		^*^	^*^	^*^	^*^		6/9
Evangelidis et al., 2006 (21)		^*^	^*^	^*^	^*^	^*^	^*^	^*^	^*^	8/9
Kadoriku et al., 2024 (22)		^*^	^*^	^*^	^*^	^*^	^*^	^*^		7/9
Kouba et al., 2024 (23)		^*^	^*^	^*^	^*^	^*^	^*^	^*^		7/9
Krafft et al., 2022 (24)		^*^	^*^	^*^	^*^	^*^	^*^	^*^		7/9
Liu et al., 2014 (25)		^*^	^*^	^*^	^*^	^*^	^*^	^*^		7/9
Alonso Mediavilla et al., 2022 (26)		^*^		^*^				^*^		3/9
Alhamdani et al., 2023 (28)		^*^	^*^	^*^	^*^	^*^	^*^	^*^		7/9
